# Expansion of human primary hepatocytes *in vitro* through their amplification as liver progenitors in a 3D organoid system

**DOI:** 10.1038/s41598-018-26584-1

**Published:** 2018-05-29

**Authors:** Delphine Garnier, Ruoya Li, Frédéric Delbos, Angélique Fourrier, Camille Collet, Christiane Guguen-Guillouzo, Christophe Chesné, Tuan Huy Nguyen

**Affiliations:** 1 INSERM, Université de Nantes, Centre de Recherche en Transplantation et Immunologie UMR 1064, Nantes, France; 2 Institut de Transplantation Urologie Néphrologie (ITUN), CHU Nantes, Nantes, France; 3 CRCINA INSERM U1232, Institut de Recherche en Santé de l’Université de Nantes, 8 quai Moncousu, Nantes, France; 4 Biopredic International, Saint-Grégoire, France

## Abstract

Despite decades of investigation on the proliferation of adult human primary hepatocytes, their expansion *in vitro* still remains challenging. To later be able to consider hepatocytes as a cell therapy alternative or bridge to liver transplantation, dramatically impeded by a shortage in liver donors, the first step is having an almost unlimited source of these cells. The banking of transplantable hepatocytes also implies a protocol for their expansion that can be compatible with large-scale production. We show that adult human primary hepatocytes when grown in 3D organoids are easily amplified, providing a substantial source of functional hepatocytes ready for transplantation. Following their plating, differentiated human hepatocytes are amplified during a transient and reversible step as liver progenitors, and can subsequently be converted back to mature differentiated hepatocytes. The protocol we propose is not only compatible with automated and high-throughput cell culture systems, thanks to the expansion of hepatocytes in suspension, but also guarantees the generation of a high number of functional cells from the same patient sample, with a relatively easy set up.

## Introduction

The liver is an essential organ that guarantees detoxification and many metabolic functions, including the synthesis of some plasma proteins, production of hormones and bile, regulation of cholesterol and glucose storage. Any liver injury can therefore have dramatic consequences, possibly leading to hepatic pathologies such as hepatitis, liver fibrosis, cirrhosis or hepatocarcinoma. Fortunately, to maintain its homeostasis the liver does possess the impressive capacity to regenerate thanks to the proliferation of mature hepatocytes in the healthy tissue^1^. However in some cases of extreme severe injury, resident stem cells may take over to reconstitute liver tissue if the unique proliferative capacity of hepatocytes is not sufficient enough to compensate for the lost^2,3^. A lot of works have addressed the question of the nature of these liver stem cells, also referred to as liver progenitor cells or oval cells, however their location and characteristics are still not well understood^4,5^.

In terms of therapy, whether the damage is induced by a virus, an oncogene, a drug or even surgical removal, the best way to treat hepatic disease when liver regeneration is inadequate remains the transplantation of the whole organ or of some fraction of the liver^6^. However the dramatic shortage of liver organs available for transplantation makes it difficult to satisfy the medical needs. To compensate for the lack of liver donors some other alternatives have to be found, including transplantation of hepatocytes^7,8^. Despite several decades of investigation and optimization of different cell culture systems, the *in vitro* amplification of hepatocytes on long-term remains still very challenging, mainly due to a lost of their differentiation features and a very poor proliferation potential in culture^9^.

Tissue engineering has received a lot of attention lately, as recent progress has opened new perspectives, for example to generate patient-specific human hepatocytes by the differentiation of pluripotent stem cells into hepatocytes^10^, either from induced pluripotent stem cells (iPS)^11–13^ or embryonic stem cells (ES)^14–16^. These new stem cells sources to generate hepatocytes-like cells are very promising but at this time there are still limitations, including the cost and time needed to maintain and differentiate cells into hepatocytes, as well as the potential genetic instability associated with the reprogramming strategy, compromising pitfall that will have to be considered with regard to clinical applications.

To reconstitute more faithfully the *in vivo* hepatic environment, 3D culture systems have also generated an increasing interest over the last decades. Few years ago Takebe *et al*. were able to generate *in vitro* some vascularized 3D liver buds resembling the adult liver tissue, by combining three cell types in the same 3D structure, that is endodermal cells derived from induced pluripotent stem (iPS), human umbilical vein endothelial cells (HUVECs) and mesenchymal stem cells (MSCs)^17^. The functionality of their system was validated by the rescue of drug-induced liver failure in mice after transplantation of human liver buds. However, when speaking about replacing liver transplantation by hepatocytes transplantation in human, that implies an almost unlimited source of mature human hepatocytes to be able to meet the needs. Despite their capacity to mimic *in vitro* liver tissues, it is difficult to consider liver buds model for the high scale production of human mature hepatocytes, as it rather favors the functionality/differentiation of liver cells than their proliferation, and requires a fastidious protocol.

On the other hand, along this line of 3D cell culture, more recently Huch *et al*. and other teams showed the long-term expansion of adult human liver cells as 3D organoids culture^18–20^. By allowing the restoration of a 3D configuration and the contact with the extracellular matrix (ECM), combined with the proper cocktail of growth factors and small molecules (EM expansion media), they managed to maintain Epcam (marker of liver progenitors) positive liver cells and induce their proliferation on long term, starting from liver tissue. A population of liver progenitors with high proliferative capacity emerged from this 3D culture, that once transferred to differentiation media (DM) became fully functional hepatocytes capable of engraftment in different mouse models^18^. This work does represent a striking progress for the generation of human adult hepatocytes, however the model may suffer of some limitations when it comes to the transfer to clinical or industrial applications^21^. In particular, the 3D organoid culture consists in growing cells inside a polymerized Matrigel drop, that by being very fragile and easily breakable poses a problem on the practical side when thinking of large-scale production.

Therefore we did investigate a new protocol to improve long term culture of human hepatocytes, and enable a transfer to industrial production thanks to a compatible process of culture. With this paper we propose a new technic to amplify human hepatocytes as 3D organoids in suspension, without the need of sorting Epcam positive cells. Besides the advantage of suspension culture, suitable for large-scale production in bioreactors, our work is a clear improvement of the previous protocol because of its increased efficiency at maturation towards functional hepatocytes, it also suggests that this cell culture system could be more stable over time.

## Results

### Human hepatocytes from different cell batches grow differently in 3D organoids

**Figure 1:**
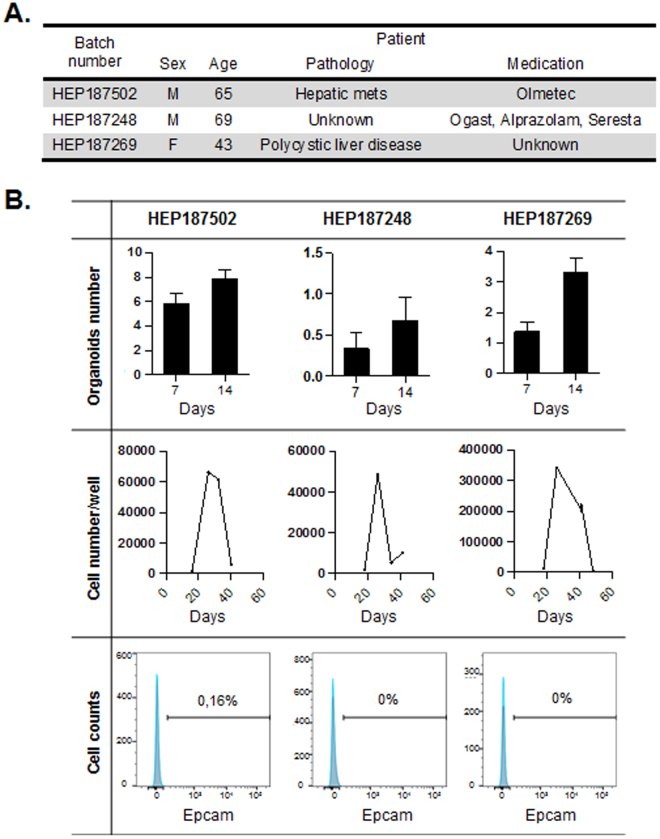
Variability in kinetic of growth of human hepatocytes when cultured as 3D organoids, depending on the cell batch. (**A**) Characteristics of human hepatocytes batches used. (**B**) Top panel shows organoids counting per well, 1 and 2 weeks after plating. Middle panel indicates the cell number production per well of a 24-wells plate over time. Cells were dissociated and counted once a week, and plated back in the same conditions. And the respective percentage of Epcam positive cells in each batch, measured by FACS, is shown in lower panel.

Starting from the protocol of Huch *et al*.^18^ to grow liver cells in 3D organoids we first tested this culture system on different batches of liver cells, except that contrary to this study we did not sort Epcam positive cells but used cryopreserved mature human hepatocytes. These cells were isolated from the healthy tissue of three different patients, each suffering from a different pathology (Fig. 1A). When analyzing the number of organoids in the three cell batches separately, it appears that there is a lot of variability between patient samples (Fig. 1B, upper panel). In parallel the cell count at different time points showed that the kinetic of proliferation differs from batch to batch, with a peak in proliferation around 30 days after plating and a decline in growth thereafter (Fig. 1B, middle panel). Of note, despite the drop in the growth rate overtime, cells did not all died by 50 days but a cell fraction still continued to proliferate for more than 2 months. In parallel the FACS analysis of the three different batches of hepatocytes showed that they are all negative for Epcam marker (Fig. 1B lower panel, Supplementary Fig. 1A and Supplementary Methods). This was also confirmed by immunofluorescence experiment on hepatocytes 24 h after plating (Supplementary Fig. 1B).

To further investigate the properties of hepatocytes growing in 3D organoids, we decided to focus on the batch HEP187269, that showed a better progression of proliferation between week 1 and 2, a higher number of cells per well and a better proliferation rate on long term (Fig. 1B). This batch was used for all the subsequent experiments.

### Human hepatocytes plated as 3D organoids acquire features of liver progenitors

We then compared the expression of different mature/progenitor liver cell markers, in short term adherent hepatocyte culture 24, 48 and 72 h after plating, and in long term 3D organoid culture around 40 days after plating and close to 2 months after plating (when cell number dramatically decreases, see Fig. 1B).

**Figure 2:**
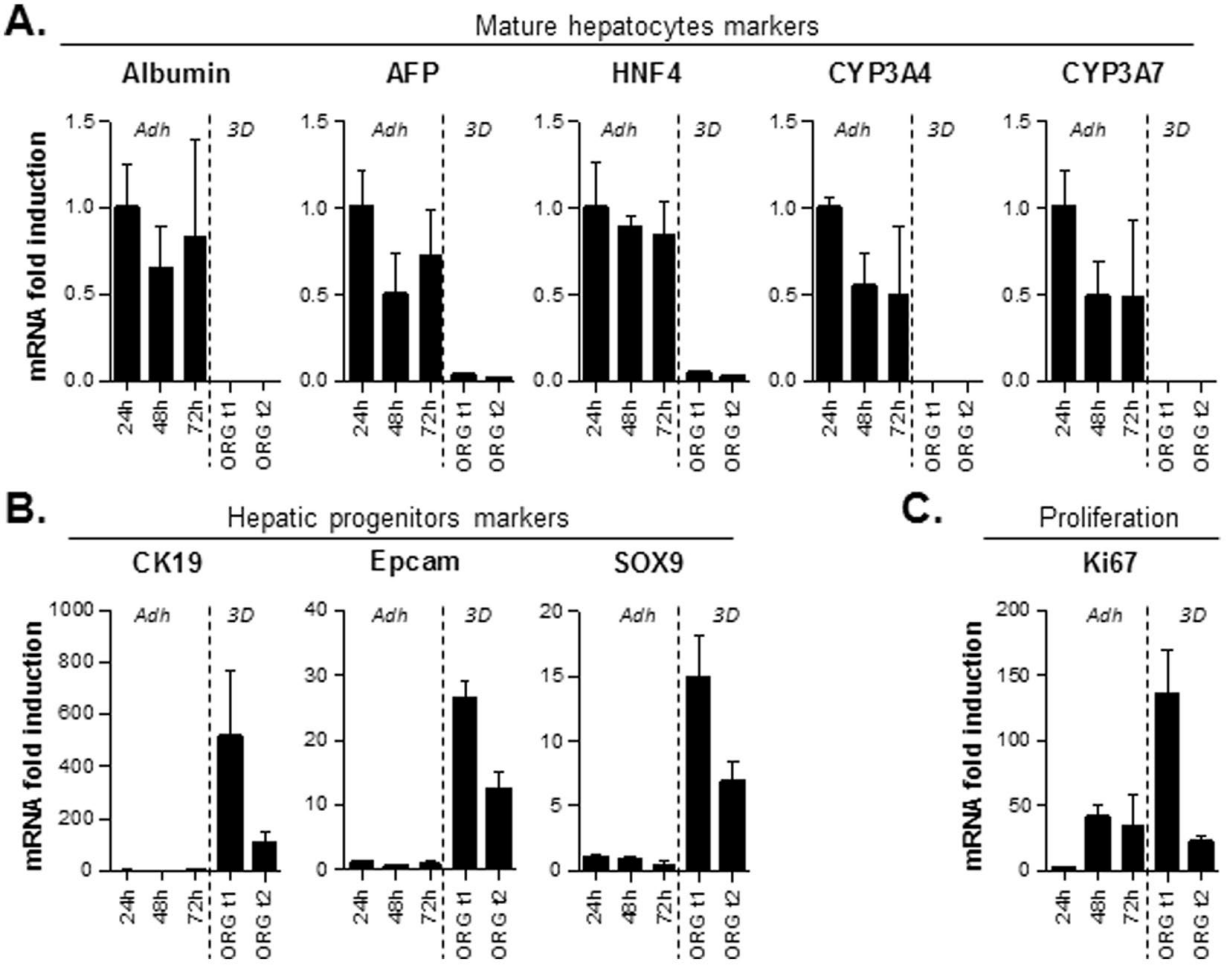
Culture of human hepatocytes as 3D organoids leads to long term survival, loss of mature hepatocyte markers, and enrichment in liver progenitors markers. (**A**) The expression of mature hepatocytes markers was analyzed by qPCR in adherent hepatocytes at 24, 48 and 72 h after plating (*Adh*), or in hepatocyte organoids (*3D*) after 2–3 passages (t1; around 40 days after plating) and 2 weeks later (t2). (**B**) The expression of markers of liver progenitors was analyzed in the same conditions, as well as Ki67 proliferation marker (**C**). The expression was normalized compared to the expression in adherent human hepatocytes 24 h post-plating. (N = 3).

**Figure 3:**
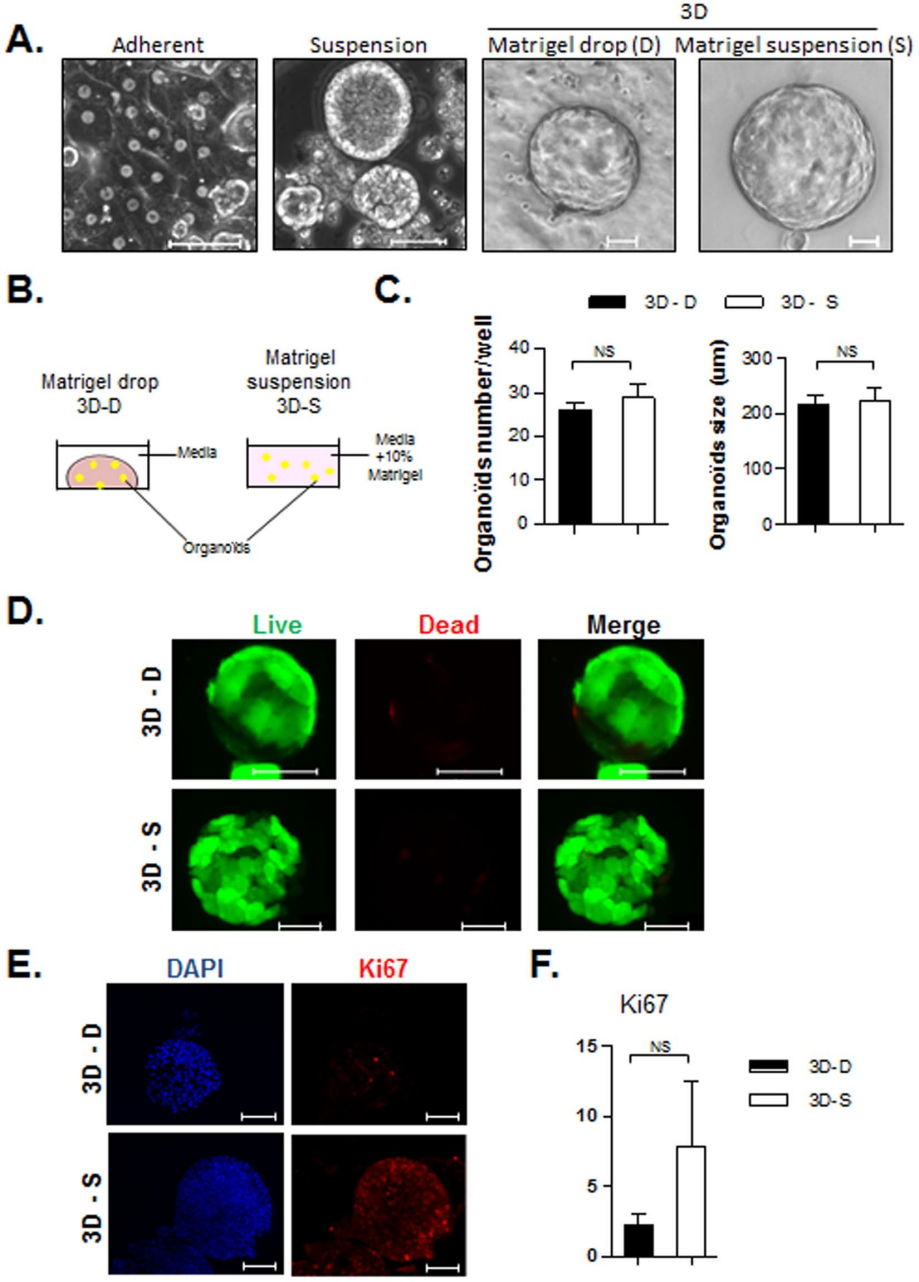
3D organoid culture of mature human hepatocytes in Matrigel drops or in Matrigel suspension display comparable levels of viability and growth on short-term. (**A**) Observation by phase contrast microscopy of human hepatocytes cultured as adherent cells, in suspension, or maintained in 3D inside Matrigel drop or in Matrigel suspension (bar graph = 50 μm). (**B**) Schematic representation of the protocol settings. (**C**) Average organoid counts 3 weeks after plating as 3D, in Matrigel drops (3D-D - black bars) or in Matrigel suspension (3D-S - white bars) (number of organoids per well of a 24-wells plate) (N = 2). (**D**) Analysis of cell viability with Live (green)/Dead (red) staining kit (bar graph = 50 μm). Proliferation was analyzed by Ki67 immunofluorescence (**E**) (bar graph = 100 μm) and qPCR (F) (N = 3).

When plated as adherent on collagen I substrate, human hepatocytes display a high level of hepatic functions, however the proliferation rate is very low and the survival limited: as early as 72 h hepatocytes start to activate apoptosis pathways and cells die by 2 weeks^9^. In parallel when liver cells are cultured as 3D organoids, the survival can be maintained on long term and an active proliferation can be obtained^18^. We did compare those two different cell culture systems, and indeed hepatocytes cultured as adherent express a high level of differentiation markers such that Albumin, CYP3A4 or CYP3A7 (Fig. 2A-*Adh*). On the contrary hepatocyte organoids maintained on long term do not express those markers or at a very low level (Figs 2A–*3D*). Interestingly that coincides with the induction of markers of hepatic progenitors, such as a 500 fold induction of Cytokeratin 19 (CK19) compared to adherent hepatocytes, 25 fold induction of Epcam or 15 fold induction of Sox9 (Fig. 2B). According to the Ki67 mRNA level, the proliferation rate is also dramatically increased in 3D organoids, with a 150 fold induction (Fig. 2C-*ORGt1*). Of note is that the second time point used in 3D culture shows a decline in proliferation as well as in the expression of progenitor markers (Fig. 2BC-*ORGt2*). Coherently with the measurement of cell number over time (Fig. 1B), there is clearly an exhaustion of organoids cell culture when getting close to 2 months cell culture.

While short term adherent culture goes with highly differentiated hepatocytes, long term 3D organoid culture is rather associated with highly proliferating progenitor cells. Plating mature human hepatocytes as 3D culture therefore leads to an enrichment in hepatic progenitors.

### Liver organoids can also be amplified in Matrigel suspension instead of Matrigel embedding

In an attempt to develop a similar 3D culture system that would not necessitate inclusion of the organoids inside polymerized Matrigel, different culture settings have been tried. Adherent culture is a well-known culture system for hepatocytes (Fig. 3A-first picture), but survival is limited to few days and proliferation is very low^9^. In our hands growing hepatocytes in suspension initially gave rise to clusters of cells that looked healthy and growing (Fig. 3A-second picture), however we were not able to dissociate the spheroids formed, and cells eventually died after few weeks. When looking at 3D systems, following Huch protocol^18^ hepatocytes formed organoids that could be maintained on long term (Fig. 3A-third picture), but interestingly similar structures could also be obtained when culturing cells in suspension in media containing some Matrigel (Fig. 3A-fourth picture). Cell culture conditions were the same in both systems, except that in the classic 3D organoids culture cells were stuck inside a drop of polymerized Matrigel, whereas in our suspension system organoids were floating in media containing 10% Matrigel (see schematic representation Fig. 3B). The measurement of organoids count and average size 3 weeks after plating did not show a significant difference between organoids grown in Matrigel drop or in Matrigel suspension (Fig. 3C). The viability inside the organoids formed was also comparable (Fig. 3D). Analysis of proliferation suggested that the growth rate could be slightly higher in Matrigel suspension compared to Matrigel drop, as indicated by the immunofluorescence experiment on the proliferation marker Ki67 (Fig. 3E). The mRNA expression of Ki67 was also increased in Matrigel suspension (Fig. 3F), however the difference was not significant. Therefore data suggest a proliferation benefit for the 3D Matrigel suspension system, further experiments will be needed to confirm it.

### Cells amplified in 3D Matrigel suspension organoids are also liver progenitors, exhibiting a low expression of differentiation markers

**Figure 4:**
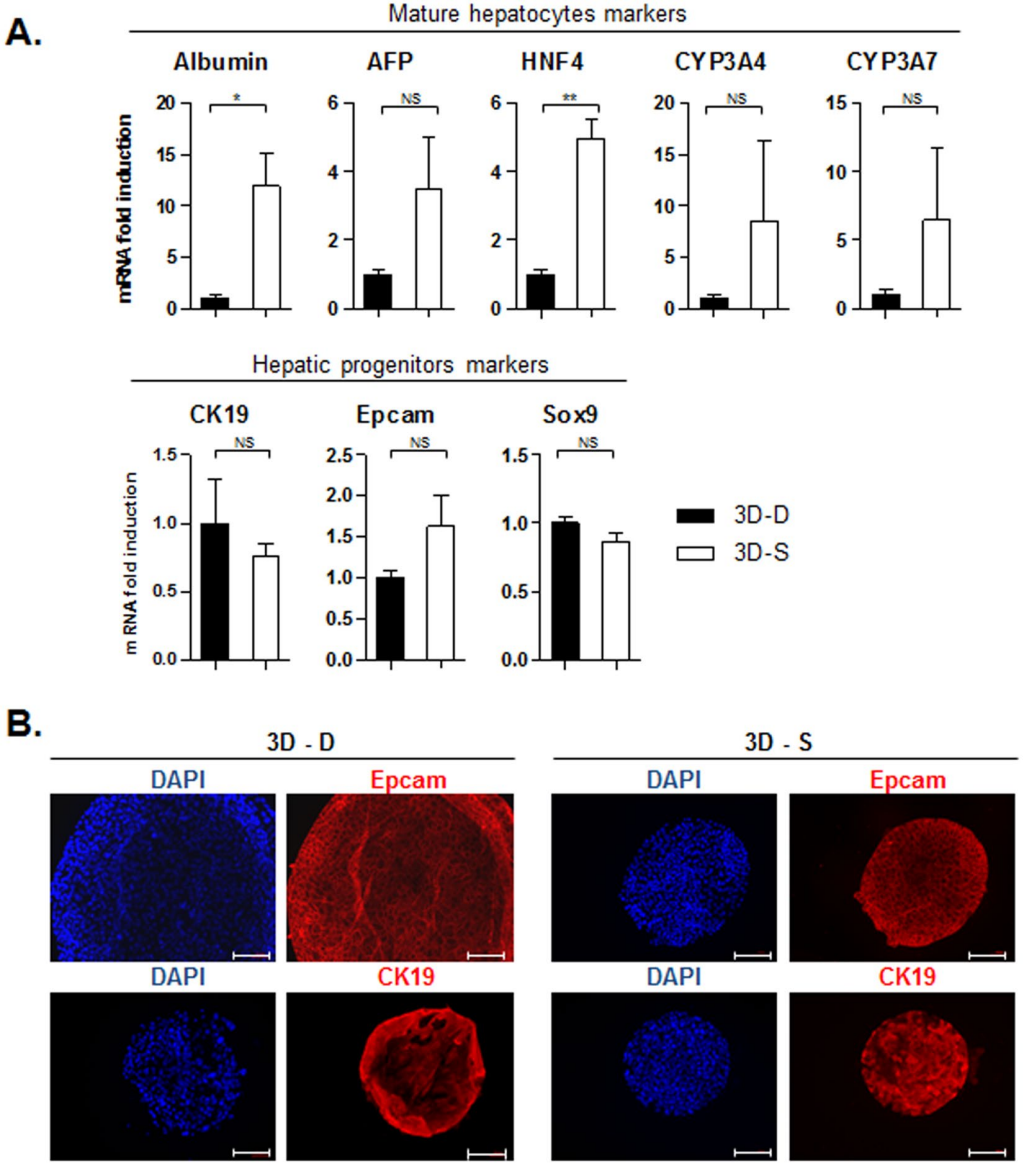
3D organoids cultured in Matrigel suspension also allows amplification of liver progenitors, while maintaining a higher level of differentiated functions. (**A**) The expression of different liver progenitors/mature hepatocyte/proliferation markers was analyzed at the mRNA level by qPCR, in 3D culture of human hepatocytes inside Matrigel drop (3D-D - black bars) or in suspension (3D-S - white bars), after 2–3 passages (t1; close to 40 days after plating) and 2 weeks later (t2). Data were normalized to the expression in Matrigel drop organoids. (N = 3) (**B**) The expression of some of those markers was validated at the protein level by immunofluorescence (bar graph = 100 μm).

To study whether plating mature hepatocytes in 3D organoid culture would lead to the amplification of the same cell type, in Matrigel drops or in Matrigel suspension, we then analysed their mRNA profiles by qPCR. As shown earlier (Fig. 2), when differentiated hepatocytes are cultured in 3D organoids in Huch & Clevers conditions (Matrigel drops)^18^, cells exhibit a high level of markers of liver precursors and lose their differentiation markers, evidencing an enrichment of a liver progenitors population. When comparing the level of expression of those markers with the one measured in 3D Matrigel suspension culture, we observed that the mRNA expression of progenitors markers is very similar (Fig. 4A-lower panel). This was confirmed by immunofluorescence experiment, that showed similar levels of staining for Epcam and CK19 in both 3D conditions (Fig. 4B). On the other hand, organoids seem to exhibit a higher level of expression of differentiation markers when cultured as 3D in Matrigel suspension (Fig. 4A-upper panel). Indeed Albumin expression was close to 12 fold higher in organoids maintained in Matrigel suspension compared to Matrigel drops, whereas HNF4 expression was increased by a factor 5. Other markers such that AFP, CYP3A4 and CYP3A7 were not significantly increased, even though they tend to show higher levels of expression in Matrigel suspension. Overall those data therefore demonstrate that whether it be in Matrigel drops or in Matrigel suspension, 3D organoids cultured in those conditions exhibit features of liver progenitors, with similar levels of expression. However, the decrease in features of mature hepatocytes observed in parallel is less pronounced in Matrigel suspension culture, that exhibit a significantly higher level of expression of differentiation markers such that Albumin and HNF4. This observation therefore suggests that if inducing these progenitors grown as 3D organoids to differentiate, to give back mature hepatocytes, Matrigel suspension culture could present an advantage.

### Organoids transferred to differentiation conditions exhibit a more mature profile when cultured in Matrigel suspension compared to the Matrigel drop system

**Figure 5:**
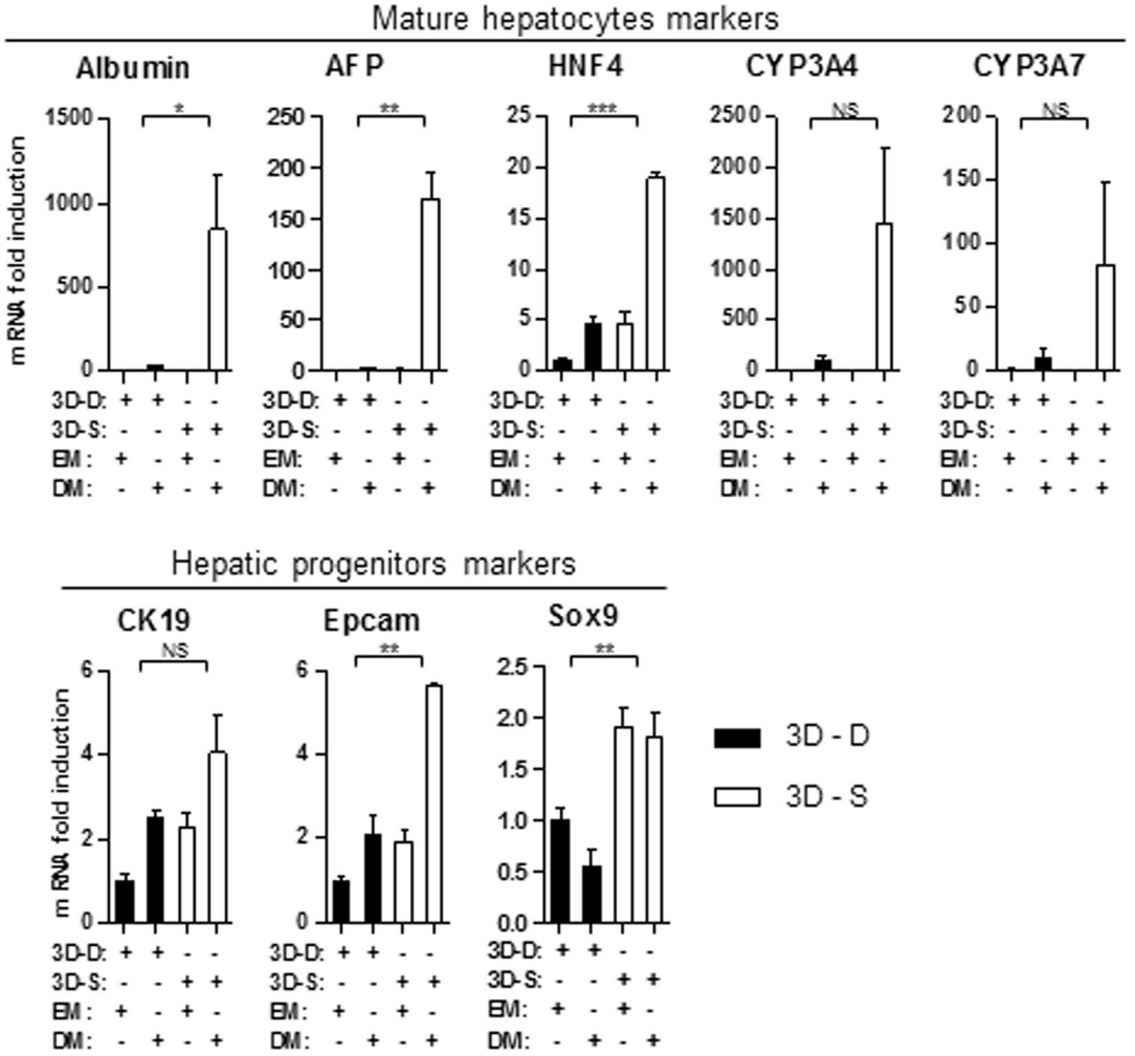
Induction of differentiation of 3D organoids into mature hepatocytes is more efficient if cultured in Matrigel suspension. The mRNA expression level of different liver progenitor/mature hepatocyte/proliferation markers was analyzed by qPCR, in 3D culture of human hepatocytes inside Matrigel drops (3D-D – black bars) or in Matrigel suspension (3D-S – white bars), in expansion (EM) or differentiation media (DM). (N = 3).

In light of the previous observation of a higher level of expression of Albumin and HNF4 in Matrigel suspension organoids, we then interrogated whether this could be correlated to a better inclination to differentiate when transferred to the DM differentiation media. Indeed after 2 weeks in differentiation media, in Matrigel suspension cells did exhibit a more than 800 times higher level of Albumin, 150 times higher level of AFP, and close to 20 times more HNF4 expression compared to culture in 3D drops (Fig. 5-upper panel). The expression of CYP3A4 and CYP3A7 seemed to be higher in Matrigel suspension too, however the difference was not significant. Surprisingly this differentiation was associated with a slight increase in the expression of the progenitors markers Epcam and Sox9 (Fig. 5-lower panel).

## Discussion

To overcome the serious shortage of donor livers the optimization of liver cell therapy is becoming essential, therefore different technologies are now developed for the generation of liver cells^7^. This includes differentiation of hepatocytes-like cells from iPS or ES cells, however some major issues remain especially with regard to their functionality, as the maturity displayed by those cells is questionable, and their genetic stability is uncertain yet^22^.

The direct expansion of adult hepatocytes from healthy tissue would therefore be the method of choice, nonetheless the culture of human mature hepatocytes *in vitro* is limited in time and is subject to a quick loss of functionality. Thanks to the 3D organoid protocol set up by Huch *et al*. in^18^, differentiated human hepatocytes can be produced in large quantity starting from the same batch of cells, avoiding variability from batch to batch and allowing a standardization of their phenotype.

Based on this model, we propose a new 3D organoid system to produce *in vitro* large quantities of human mature hepatocytes starting from a low number of cryopreserved cells, with a protocol of culture in suspension allowing an easy cell passaging and large-scale amplification.

Of note is the fact that whereas Huch *et al*. started from EpCAM positive cells, we did first start from human hepatocytes as fully differentiated hepatocytes. While they grew in 3D in expansion medium, the cells started to exhibit a phenotype of liver progenitors with a high proliferation capacity (Fig. 2). This was paralleled by a decrease in hepatocyte-specific functions, which is reversible as cells reacquire a differentiated phenotype when they are switched to the DM maturation media (Fig. 5). Surprisingly in contrast to Huch *et al*. who claimed that the Epcam negative hepatocyte fraction can not generate organoids, our results show that purified hepatocytes are able to develop into organoids with features of liver progenitors (Fig. 2). Their expression of SOX9, CK19 and EpCAM however attest of their ductal origin, asking the question of the origin of these cells. Several hypotheses could explain this discrepancy. One cannot exclude the presence of some very rare EpCAM positive ductal contaminant cells that took over the prevalent hepatocyte population. The uncovered plasticity of mature hepatocytes also allows us to consider some dedifferentiation into liver progenitors, as revealed by several previous works^23–26^. In fact a recent work demonstrated that rat terminally differentiated hepatocytes can be converted into bipotent liver progenitors, also called chemically induced liver progenitors (CLiPs), with some of the chemicals included in the EM media^27^. When looking at the number of cells amplified, that would on the other hand mean that the dedifferentiation process would concern only a fraction of the initial plated population.

The question whether the amplified cells were directly converted from differentiated hepatocytes or from some underlying precursors cells will be further investigated, it still remains from our observations that starting from purified human hepatocytes we can generate a progenitor population with an elevated proliferation potential, while keeping the capacity to differentiate into mature hepatocytes. This is of considerable interest when considering the relatively easy procedure, and the great benefit of having the capacity of producing a big quantity of hepatocytes from short supply. To add on to the breakthrough made by Huch *et al*.^18^, which was to introduce 3D organoid expansion of liver progenitors, our results therefore suggest that an even more accessible source of hepatic cells would be frozen purified hepatocytes, commercially available and without the need of FACS-sorting EpCAM positive cells or using freshly isolated cells.

Our major finding is the demonstration that, when culturing 3D organoids in a suspension media that contains diluted Matrigel instead of embedded inside a pure Matrigel drop, culture conditions can be really improved to obtain 1- a more efficient differentiation, 2- a process compatible with production in bioreactors, and 3- potentially a higher rate of proliferation and a better stability on long term.

First using Huch *et al*. differentiation media we report that cells maintained as 3D organoids in Matrigel suspension, while showing features of progenitors, preserve a higher level of expression of differentiation markers. That results in a more efficient conversion into mature hepatocytes when shifted to DM media, compared to 3D organoid in Matrigel drops (Fig. 5). This constitutes a remarkable advantage, as this new procedure not only could save time and money on hepatocytes production, but those observations also suggest that in the context of transplantation cells would most probably be more prone to finish their maturation *in vivo*, leading to optimized functionality of transplanted cells and rapidity of response in case of acute liver injury.

Secondly, on a practical aspect we designed an adaptation of the 3D liver organoids culture that is suitable on a large-scale, thanks to the suspension culture. Indeed this attribute makes the process adaptable for the culture inside bioreactors in large volumes of media. Importantly, while the 10% Matrigel suspension culture gave rise to organoids with a similar morphology and phenotype compared to Matrigel drop culture, this was not the case for classical suspension culture. By comparison the same hepatocytes incubated in suspension in the EM media, devoid of Matrigel, never formed organoids that could be maintained in culture, and rather ended up in aggregates (Fig. 3A- second picture) that eventually died. This difference may be explained by the crucial role played by the contact with the ECM, provided by the presence of Matrigel in the media^28^.

Finally, when looking at the proliferation activity in cell culture, over time 3D organoids experience a decrease in proliferation (Fig. 1). They can be maintained in culture for several months, however their growth rate declines overtime. Similarly Huch *et al*. also observed a prolongation of the cell doubling time over time. Our preliminary data will have to be confirmed, especially on long term culture, but they suggest that the proliferation index would be maintained at a higher level over time when organoids are cultured in Matrigel suspension compared to Matrigel drop 3D culture (Fig. 3), offering a potential further optimization of the system by delaying this cellular exhaustion observed in culture.

In summary, we hope these findings will be beneficial for the research on liver failure treatment, by providing a new way to generate large quantities of mature hepatocytes, after an intermediary step of expansion as bipotent progenitors growing in suspension. This protocol presents the advantage of using some easily accessible starting material, that is cryopreserved hepatocytes without FACS sorting. Finally, converting a system previously established as 3D solid polymer into a suspension culture makes it suitable for the production of hepatocytes in large containers and cell culture automation.

As a perspective for a clinical use, this *in vitro* matrigel-expanded hepatocytes could be used in the context of external bioartificial liver devices with encapsulation, as some bioartificial liver devices with hepatic cells have been safely used for treating patients with end-stage liver diseases^29–32^.

## Methods

### Cell culture

#### Cryoplateable primary human hepatocytes

Cryopreserved primary human hepatocytes were provided by Biopredic, and thawed according to the manufacturer instructions. Briefly, human primary hepatocytes were isolated from tumor-free margin of the resected liver tissue by a two-step collagenase perfusion technique. After washing steps, the obtained hepatocytes were purified by Percoll density gradient and then cryopreserved using an optimized freezing process. After thawing, cells were plated on Collagen I-coated plates, and maintained in William’s E GlutaMAX^TM^ medium (Life Technologies) supplemented with 4 μg/mL Bovine Insulin (Sigma) and 50 μM Hydrocortisone (Sigma). Details of hepatocytes batches are indicated in Fig. 1.

#### Culture as 3D organoids in Matrigel drops (3D-D)

Primary hepatocytes were cultured as 3D organoids according to the protocol set up by Huch and Clevers^18^. Briefly, after centrifugation primary hepatocytes were mixed with growth factor reduced Matrigel (Corning), and a 50 μL drop of this solution was plated in the middle of each well of a 24-well plate. For the first set of experiments, comparing organoïds formation in three different batches of human hepatocytes (Fig. 1), 30,000 cells per well have been plated. For the other experiments, focusing on HEP187269, 300,000 cells per well were used. After solidification of Matrigel the EM expansion media was added, composed of AdDMEM/F12 (Life Technologies) supplemented with 1% N2 and 1% B27 (Life technologies), 1.25 mM N-Acetylcysteine (Sigma), 10 nM gastrin (Sigma), 10 mM Nicotinamide (Sigma), 5 μM A83.01 (Tocris), 10 μM FSK (Tocris), and the growth factors: 50 ng/mL EGF (R&D Systems), 500 ng/mL Rspo1 (Peprotech), 100 ng/mL FGF10 (Miltenyi Biotec), 25 ng/mL HGF (Miltenyi Biotec). During the 3 first days after plating, the media was supplemented with 25 ng/mL Noggin (Peprotech), 50 ng/mL Wnt3a (R&D Systems), and 10 μM Y27632 (Stem Cell Technologies).

#### Culture as 3D organoids in Matrigel suspension (3D-S)

Alternatively primary hepatocytes were cultured as 3D organoids in 10% Matrigel suspension, in similar conditions of culture except organoids were floating in Huch and Clevers EM media^18^ containing 10% of growth factors-reduced Matrigel, instead of embedded in a Matrigel drop. After dissociation of organoids the cell suspension was plated on Ultra-Low Attachment plates (Corning).

#### Differentiation of organoids into hepatocytes

Differentiation of organoids into hepatocytes was induced as previously described^18^. Briefly, after amplification organoids were first cultured in EM media supplemented by BMP7 (25 ng/ml) for 7–10 days, then split and plated back in the same media for 2–4 days. Organoids were subsequently switched to the DM differentiation media, composed as follows: AdDMEM/F12 medium (Life Technologies), 1% N2 and 1% B27 (Life Technologies), 50 ng/mL EGF (R&D Systems), 10 nM gastrin (Sigma), 25 ng/mL HGF (Miltenyi Biotec), 100 ng/mL FGF19 (R&D Systems), 500 nM A83.01 (Tocris), 10 μM DAPT (Sigma), 25 ng/mL BMP7 (Peprotech), and 30 μM Dexamethasone (Sigma).

### RNA isolation and Real-time RT-PCR

Total RNA was isolated and purified from cells using the RNeasy Mini Kit (Qiagen). Real-time reverse-transcription was performed starting from 5 ng RNA, with a one-step RT-PCR kit using Taqman technology (AgPath-ID™ One-Step RT-PCR, Life Technologies) and the specific following probes: Albumin Hs00910225_m1; AFP Hs00173490_m1; HNF4A Hs00230853_m1; CYP3A4 Hs00604506_m1; CYP3A7 Hs00426361_m1; Cytokeratin 19 Hs00761767_s1; Epcam Hs00901885_m1; SOX9 Hs00165814_m1; Ki67 Hs00267195_m1)(Life Technologies) and using the Applied Biosystems ViiA 7 Real-Time PCR System. GAPDH was used as a housekeeping gene. Results were normalized as specified in figures legends.

### Immunocytochemistry

Cells were fixed in 4% PFA. After permeabilization in 0.5% Triton X-100, cells were blocked with 1% BSA and incubated overnight at 4 °C with the following primary antibodies: goat anti-Epcam antibody (R&D Systems), mouse anti-CK19 (Dako) or rabbit anti-Ki67 (Abcam). Secondary antibodies were then incubated for 1 h at room temperature (Life Technologies), and Prolong Gold mounting media with DAPI (Life Technologies) was used for mounting on a glass slide.

### Live/Dead staining kit

Viability on cells was assessed by using the Live/Dead Viability/Cytotoxicity Kit for mammalian cells (ThermoFisher Scientific), according to the manufacturer instructions. Briefly, a mix of 2 µM calcein AM and 4 µM EthD-1 was prepared and incubated with cells for 45 min at room temperature, the preparation was then observed under a Zeiss fluorescent microscope.

### Statistical analysis

All results are presented as number of replicates (N) and mean value of replicates ± SD. Statistical analysis was performed using two-tailed unpaired t-test with GraphPad Prism software (GraphPad, USA).

## Electronic supplementary material

Supplementary information
