# Supplementary material for: Expansion of human primary hepatocytes in vitro through their amplification as liver progenitors in a 3D organoid system
Source: Sci Rep. 2018 May 29;8:8222. doi: 10.1038/s41598-018-26584-1 (PMC5974235; doi:10.1038/s41598-018-26584-1)
Supplement: Supplementary file 1 — Supplementary information [file 41598_2018_26584_MOESM1_ESM.pdf]

Expansion of human primary hepatocytes in vitro through their amplification as liver progenitors in a 3D organoid system

Delphine Garnier, Ruoya Li, Frédéric Delbos, Angélique Fourrier, Camille Collet, Christiane Guguen-Guillouzo, Christophe Chesné, Tuan Huy Nguyen

Supplementary data

A.

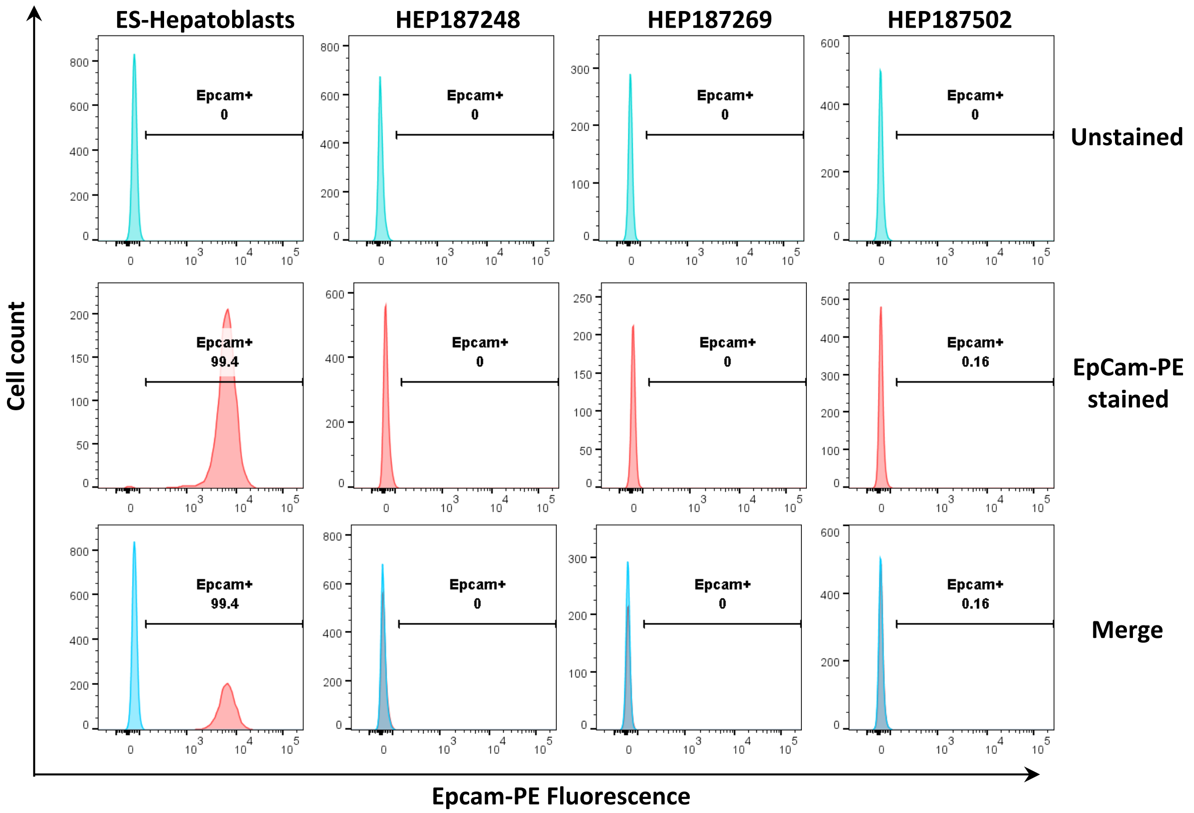

B.

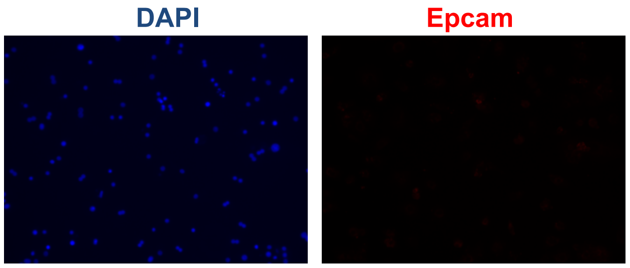

Supplementary Figure 1

Cryopreserved human hepatocytes are negative for Epcam expression, marker of liver progenitors. (A) The expression of Epcam in the three different batches of human hepatocytes tested was analyzed by FACS before plating them in 3D culture. Hepatoblasts derived from human embryonic stem cells were used as a positive control (left panel). Fluorescence intensities for non stained (top panel), and Epcam-PE stained (middle panel) cells are shown, as well as merged plot (bottom panel). No Epcam positive cells were detected in batches HEP187248 and HEP187269, whereas only a negligible fraction was detected in batch HEP187502. (B) The absence of expression of Epcam in primary hepatocytes was confirmed by immunostaining.

Supplementary Methods

Flow cytometry

Primary human hepatocytes were resuspended in PBS and 2.10^5 cells per condition were incubated with viability dye eFluor 450 for 45min (eBioscience), then 30min with fixation/permeabilization buffer (eBioscience), 15min with 2% Fetal Calf Serum and 30min with PE Mouse Anti-Human EpCAM antibody (BD Biosciences). Cells were then washed several times, fixed with paraformaldehyde 2% and analyzed with a FACSCanto II flow cytometer (BD Biosciences). As a positive control for Epcam expression, hepatoblasts derived from human embryonic stem cells after 11 days of differentiation were used. Data were analyzed and plotted using FlowJo software.

Production of human embryonic stem cell-derived hepatoblasts

The ESI017 cell line (Biotime) was cultured in wells pre-coated with 5 µg/mL laminin 521 (Biolamina) in mTeSR1 medium (Stemcell Technologies). Endoderm differentiation was initiated at 30-40% confluence by replacing mTeSR1 with RPMI 1640 medium supplemented with B27 serum-free supplement (Life technologies)(RPMI/B27), 100 ng/mL Activin A (Miltenyi Biotec), 50 ng/mL Wnt3A (R&D systems). The medium was changed every day for 5 days, except no Wnt3A was added in the medium for the last 3 days. On day 6, Hepatoblast differentiation was initiated by culturing the cells in RPMI/B27 supplemented with 10 ng/mL fibroblast growth factor (FGF) 10 (Miltenyi Biotec) and 10 ng/mL bone morphogenetic protein (BMP) 4 (R&D systems,) for 5 days with daily medium change.
